# Pharmacoproteomic study of the effects of chondroitin and glucosamine sulfate on human articular chondrocytes

**DOI:** 10.1186/ar3077

**Published:** 2010-07-13

**Authors:** Valentina Calamia, Cristina Ruiz-Romero, Beatriz Rocha, Patricia Fernández-Puente, Jesús Mateos, Eulàlia Montell, Josep Vergés, Francisco J Blanco

**Affiliations:** 1 Osteoarticular and Aging Research Lab, Proteomics Unit, Lab of Proteo-Red. Rheumatology Division, INIBIC-CHU A Coruña, As Xubias s/n, A Coruña 15006, Spain; 2 Pharmacological Research Area, Scientific Medical Department. Bioibérica S.A., Plaza Francesc Macià 7, Barcelona 08029, Spain

## Abstract

**Introduction:**

Chondroitin sulfate (CS) and glucosamine sulfate (GS) are symptomatic slow-acting drugs for osteoarthritis (OA) widely used in clinic. Despite their widespread use, knowledge of the specific molecular mechanisms of their action is limited. The aim of this work is to explore the utility of a pharmacoproteomic approach for the identification of specific molecules involved in the pharmacological effect of GS and CS.

**Methods:**

Chondrocytes obtained from three healthy donors were treated with GS 10 mM and/or CS 200 μg/mL, and then stimulated with interleukin-1β (IL-1β) 10 ng/mL. Whole cell proteins were isolated 24 hours later and resolved by two-dimensional electrophoresis. The gels were stained with SYPRORuby. Modulated proteins were identified by matrix-assisted laser desorption/ionization time-of-flight (MALDI-TOF/TOF) mass spectrometry. Real-time PCR and Western blot analyses were performed to validate our results.

**Results:**

A total of 31 different proteins were altered by GS or/and CS treatment when compared to control. Regarding their predicted biological function, 35% of the proteins modulated by GS are involved in signal transduction pathways, 15% in redox and stress response, and 25% in protein synthesis and folding processes. Interestingly, CS affects mainly energy production (31%) and metabolic pathways (13%), decreasing the expression levels of ten proteins. The chaperone GRP78 was found to be remarkably increased by GS alone and in combination with CS, a fact that unveils a putative mechanism for the reported anti-inflammatory effect of GS in OA. On the other hand, the antioxidant enzyme superoxide dismutase 2 (SOD2) was significantly decreased by both drugs and synergistically by their combination, thus suggesting a drug-induced decrease of the oxidative stress caused by IL-1β in chondrocytes.

**Conclusions:**

CS and GS differentially modulate the proteomic profile of human chondrocytes. This pharmacoproteomic approach unravels the complex intracellular mechanisms that are modulated by these drugs on IL1β-stimulated human articular chondrocytes.

## Introduction

Osteoarthritis (OA) is becoming increasingly prevalent worldwide because of the combination of an aging population and growing levels of obesity. Despite the increasing number of OA patients, treatments to manage this disease are limited to controlling pain and improving function and quality of life while limiting adverse events [1]. Effective therapies to regenerate damaged cartilage or to slow its degeneration have not been developed.

The failure of conventional treatments (analgesics or non-steroidal anti-inflammatory drugs) to satisfactorily control OA progression, combined with their frequent adverse side effects, may explain the increasing use of such SYSADOA (SYmptomatic Slow-Acting Drugs for Osteoarthritis) therapies as glucosamine sulfate (GS) and chondroitin sulfate (CS). Different clinical trials have proved that GS [2-4] and CS [5,6] are effective in relieving the symptoms of OA [7], probably due to their anti-inflammatory properties. However, although these reports were intended to resolve and clarify the clinical effectiveness of these supplements regarding OA, they leave doubts among the scientific community and fuel the controversy [8]. The recently published results of the Glucosamine/chondroitin Arthritis Intervention Trial (GAIT) showed that, in the overall group of patients with osteoarthritis of the knee, GS and CS alone or in combination did not reduce pain effectively [9]. For a subset of participants with moderate-to-severe knee pain, however, GS combined with CS provide statistically significant pain relief compared with placebo. One possible explanation for this discrepancy may be the relative participation of inflammatory cytokines in different subpopulations; and it is also hypothesized that the effects of GS and CS are better realized in patients with more severe OA, which have greater involvement of interleukin-1beta (IL-1β) [10].

With the aim to describe more clearly the effects of GS and CS on cartilage biology and characterize their mechanism of action, we performed proteomic analyses of articular chondrocytes treated with exogenous GS and/or CS. Most previous studies have evaluated single proteins, but have not addressed the total chondrocyte proteome. With the introduction of proteomics, it has become possible to simultaneously analyze changes in multiple proteins. Proteomics is a powerful technique for investigating protein expression profiles in biological systems and their modifications in response to stimuli or particular physiological or pathophysiological conditions. It has proven to be a technique of choice for study of modes of drug action, side-effects, toxicity and resistance, and is also a valuable approach for the discovery of new drug targets. These proteomic applications to pharmacological issues have been dubbed pharmacoproteomics [11]. Currently, many proteomic studies use two-dimensional electrophoresis (2-DE) to separate proteins [12]; we have recently used this proteomic approach to describe the cellular proteome of normal and osteoarthritic human chondrocytes in basal conditions [13,14] and also under IL-1β stimulation [15].

To more clearly define the effects of GS and CS on cartilage biology, we performed proteomic analyses of articular chondrocytes treated with exogenous GS and/or CS. Because the treatment efficacy of these compounds appears to vary with the pathological severity of OA, we used an *in vitro *model employing normal human chondrocyte cultures stimulated with IL-1β, a proinflammatory cytokine that acts as a mediator to drive the key pathways associated with OA pathogenesis [16].

## Materials and methods

### Reagents, chemicals and antibodies

Culture media and fetal calf serum (FCS) were obtained from Gibco BRL (Paisley, UK). Culture flasks and plates were purchased from Costar (Cambridge, MA, USA). Two-dimensional electrophoresis materials (IPG buffer, strips, and so on) were purchased from GE Healthcare (Uppsala, Sweden). IL-1β was obtained from R&D Systems Europe (Oxford, UK). Glucosamine sulfate and chondroitin sulfate were provided by Bioiberica (Barcelona, Spain). Antibody against human SOD2 was obtained from BD Biosciences (Erembodegem, Belgium), antibody against α-Tubulin from Sigma-Aldrich (St. Louis, MO, USA), antibody against human GRP78 and the correspondent peroxidase-conjugated secondary antibodies from Santa Cruz Biotechnology (Santa Cruz, CA, USA). Unless indicated, all other chemicals and enzymes were obtained from Sigma-Aldrich.

### Cartilage procurement and processing

Macroscopically normal human knee cartilage from three adult donors (44, 51 and 62 years old) with no history of joint disease was provided by the Tissue Bank and the Autopsy Service at Complejo Hospitalario Universitario A Coruña. The study was approved by the Ethics Committee of Galicia, Spain. Cartilage was processed as previously described [13].

### Primary culture of chondrocytes

Chondrocytes were recovered and plated in 162-cm^2 ^flasks in DMEM supplemented with 100 units/mL penicillin, 100 μg/mL streptomycin, 1% glutamine and 10% FCS. The cells were incubated at 37°C in a humidified gas mixture containing 5% CO_2 _balanced with air. At confluence cells were recovered from culture flasks by trypsinization and seeded onto 100 mm culture plates (2 × 10^6 ^per plate) for proteomic studies or six-multiwell plates (5 × 10^5 ^per well) for further analysis (RNA/protein extraction). Chondrocytes were used at Week 2 to 3 in primary culture (P1), after making them quiescent by incubation in a medium containing 0.5% FCS for 24 h. Verification of cell type was carried out by positive immunohistochemistry to type II collagen. Finally, cells were cultured in FCS-free medium containing glucosamine sulfate (10 mM) and/or chondroitin sulfate (200 μg/mL). Two hours later, IL-1β was added at 10 ng/ml to the culture medium. All the experiments were carried out for 24 hours. Cell viability was assessed by trypan blue dye exclusion.

### Two-dimensional gel electrophoresis (2-DE)

The 2-DE technique used in this study has been previously described [13]. Briefly, 200 μg of protein extracts were applied to 24 cm, pH 3-11 NL, IPG strips by passive overnight rehydration. The first dimension separation, isoelectric focusing (IEF), was performed at 20°C in an IPGphor instrument (GE Healthcare) for a total of 64,000 Vhr. The second dimension separation was run on an Ettan DALT six system (GE Healthcare) after equilibration of the strips. Electrophoresis followed the technique of Laemmli [17], with minor modifications. We used 1X Tris-glycine electrophoresis buffer as the lower buffer (anode) and 2X Tris-glycine as the upper buffer (cathode).

### Protein staining

Gels were fixed and stained overnight with SYPRORuby (Invitrogen, Carlsbad, CA, USA), according to the manufacturer's protocol. After image acquisition and data analysis, 2-DE gels were stained either with Coomassie Brilliant Blue (CBB) or silver nitrate according to standard protocols [18] to allow subsequent mass spectrometry (MS) identification.

### 2-DE image acquisition and data analysis

SYPRO-stained gels were digitized using a CCD camera (LAS 3000 imaging system, Fuji, Tokyo, Japan) equipped with a blue (470 nm) excitation source and a 605DF40 filter. CBB and silver stained gels were digitized with a densitometer (ImageScanner, GE Healthcare). Images from SYPRO-stained gels were analyzed with the PDQuest 7.3.1 computer software (Bio-Rad, Hercules, CA, USA).

### Mass spectrometry (MS) analysis

The gel spots of interest were manually excised and transferred to microcentrifuge tubes. Samples selected for analysis were in-gel reduced, alkylated and digested with trypsin according to the method of Sechi and Chait [19]. The samples were analyzed using the Matrix-assisted laser desorption/ionization (MALDI)-Time of Flight (TOF)/TOF mass spectrometer 4800 Proteomics Analyzer (Applied Biosystems, Framingham, MA, USA) and 4000 Series Explorer™ Software (Applied Biosystems). Data Explorer version 4.2 (Applied Biosystems) was used for spectra analyses and generating peak-picking lists. All mass spectra were internally calibrated using autoproteolytic trypsin fragments and externally calibrated using a standard peptide mixture (Sigma-Aldrich). TOF/TOF fragmentation spectra were acquired by selecting the 10 most abundant ions of each MALDI-TOF peptide mass map (excluding trypsin autolytic peptides and other known background ions).

### Database search

The monoisotopic peptide mass fingerprinting data obtained by MS and the amino acid sequence tag obtained from each peptide fragmentation in MS/MS analyses were used to search for protein candidates using Mascot version 1.9 from Matrix Science [20]. Peak intensity was used to select up to 50 peaks per spot for peptide mass fingerprinting, and 50 peaks per precursor for MS/MS identification. Tryptic autolytic fragments, keratin- and matrix-derived peaks were removed from the dataset used for the database search. The searches for peptide mass fingerprints and tandem MS spectra were performed in the Swiss-Prot release 53.0 [21] and TrEMBL release 37.0 [22] databases. Identifications were accepted as positive when at least five peptides matched and at least 20% of the peptide coverage of the theoretical sequences matched within a mass accuracy of 50 or 25 ppm with internal calibration. Probability scores were significant at *P *< 0.01 for all matches. The intracellular localization of the identified proteins was predicted from the amino acid sequence using the PSORT II program [23].

### Western blot tests

One-dimensional Western blot analyses were performed utilizing standard procedures. Briefly, 30 μg of cellular proteins were loaded and resolved using standard 10% SDS-polyacrylamide gel electrophoresis (SDS-PAGE). The separated proteins were then transferred to polyvinylidene fluoride (PVDF) membranes (Immobilon P, Millipore Co., Bedford, MA, USA) by electro-blotting and probed with specific antibodies against SOD2 (1:1000), GRP78 (1:500), and the housekeeping control α-tubulin (1:2000). Immunoreactive bands were detected by chemiluminescence using corresponding horseradish peroxidase (HRP)-conjugated secondary antibodies and enhanced chemiluminescence (ECL) detection reagents (GE Healthcare), then digitized using the LAS 3000 image analyzer. Quantitative changes in band intensities were evaluated using ImageQuant 5.2 software (GE Healthcare).

### Real-time PCR assays

Total RNA was isolated from chondrocytes (5 × 10^5 ^per well) using Trizol Reagent (Invitrogen, Carlsbad, CA, USA), following the manufacturer's instructions. cDNA was synthesized from 1 μg total RNA, using the Transcriptor First Strand cDNA Synthesis Kit (Roche Applied Science, Indianapolis, IN, USA) in accordance with the manufacturer's instructions, and analyzed by quantitative real-time PCR. Quantitative real-time PCR assay was performed in the LightCycler 480 instrument (Roche Applied Science) using 96-well plates. Primers for SOD2, GRP78 and the housekeeping genes, HPRT1 and RPLP0, were designed using the Universal Probe Library tool from the Roche website [24]. Primer sequences were as follows: SOD2 forward, 5'-CTGGACAAACCTCAGCCCTA-3'; SOD2 reverse, 5'-TGATGGCTTCCAGCAACTC-3'; GRP78 forward, 5'-GGATCATCAACGAGCCTACG-3'; GRP78 reverse, 5'-CACCCAGGTCAAACACCAG-3'; HPRT1 forward, 5'-TGACCTTGATTTATTTTGCATACC-3'; HPRT1 reverse, 5'-CGAGCAAGACGTTCAGTCCT-3'; RPLP0 forward, 5'-TCTACAACCCTGAAGTGCTTGAT-3', PRPL0 reverse 5'-CAATCTGCAGACAGACACTGG-3'. The results were analyzed using the LightCycler 480 software release 1.5.0 (Roche), which automatically recorded the threshold cycle (Ct). An untreated cell sample (basal) was used as the calibrator; the fold change for this sample was 1.0. Target gene Ct values were normalized against HPRT1 and RPLP0. Data were analyzed using the 2^-ΔΔCt ^method and expressed as fold change of the test sample compared to the basal condition [25].

### Statistical analysis

Each experiment was repeated at least three times. The statistical significance of the differences between mean values was determined using a two-tailed *t*-test. *P *≤ 0.05 was considered statistically significant. In the proteomic analysis, normalization tools and statistical package from PDQuest software (Bio-Rad) were employed. Where appropriate, results are expressed as the mean ± standard error.

## Results

**Figure 1:**
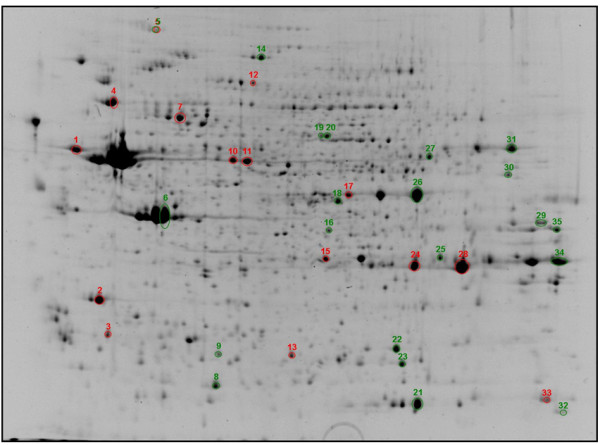
**Representative two-dimensional electrophoresis (2-DE) map of human articular chondrocyte proteins obtained in this work**. Proteins were resolved in the 3 to 11 (non linear) pH range on the first dimension, and on 10% T gels on the second dimension. The 35 mapped and identified spots are annotated by numbers according to Table 1.

To assess the influence of GS and CS on the intracellular pathways of human particularcz chondrocytes, we compared five different conditions: cells before treatment (basal), IL-1β-treated cells (control), IL-1β + GS-treated cells, IL-1β + CS-treated cells and IL-1β + GS + CS-treated cells. Two-dimensional electrophoresis (2-DE) gels of each condition were obtained from three healthy donors (a representative image of them is shown in Figure 1). The 15 digitalized images of these gels were analyzed using PDQuest analysis software. The program was able to detect more than 650 protein spots on each gel. The matched spots (540) were analyzed for their differential abundance. After data normalization, 48 protein spots were found to be altered more than 1.5-fold in the GS- and CS-treated samples (both increased and decreased compared to control condition), considering only those with a significance level above 95% by the Student's *t*-test (*P *< 0.05). These spots were excised from the gels and analyzed by MALDI-TOF and MALDI-TOF/TOF MS. The resulting protein identifications led to the recognition of 35 spots corresponding to 31 different proteins that were modulated by GS- or CS- treatment. Interestingly, some of these proteins, such as heat shock protein beta-1 (HSPB1) or alpha enolase (ENOA) were present in more than one spot, indicating that they undergo posttranslational modifications, such as glycosylation or phosphorylation. Table 1 summarizes the differentially expressed proteins identified in this proteomic analysis.

**Table 1:** Human articular chondrocyte proteins modified by treatment with interleukin-1β (IL-1β) plus glucosamine and/or chondroitin sulfate

Spot n°		Protein name	**Acc. n°**^**§**^	**GS**^**‡**^	**CS**^**‡**^	**GS+CS**^**‡**^	Loc.**	***M***_**r**_**/p*I***^**§§**^	Cellular role
1	**PDIA1**	**Protein disulfide-isomerase precursor**	**P07237**	6.54	5.60	11.24	ER, CM	57.1/4.76	Protein folding
2	**ANXA5**	**Annexin A5**	**P08758**	1.97	1.30	1.52	C	35.9/4.94	Signal transduction
3	**GDIR**	**Rho GDP-dissociation inhibitor 1**	**P52565**	2.62	1.03	2.51	C	23.2/5.03	Signal transduction
4	**GRP78**	**78 kDa glucose-regulated protein precursor**	**P11021**	8.08	1.19	14.15	ER	72.3/5.07	Protein folding
5	**CO6A1**	**Collagen alpha-1(VI) chain precursor**	**P12109**	4.14	-1.96	-1.5	EXC	108.5/5.26	Cell adhesion
6	**ACTB**	**Actin, cytoplasmic 1**	**P60709**	-3.7	-1.41	-1.89	C, CK	41.7/5.29	Cell motion
7	**HSP7C**	**Heat shock cognate 71 kDa protein**	**P11142**	7.20	3.90	5.46	C	70.9/5.37	Protein folding
8	**GSTP1**	**Glutathione S-transferase P**	**P09211**	-1.2	-1.54	-1.49	C	23.3/5.43	Detoxification
9	**HSPB1**	**Heat shock protein beta-1**	**P04792**	-1.33	-1.35	-1.75	C, N	22.8/5.98	Stress response
10	**PDIA3**	**Protein disulfide-isomerase A3 precursor**	**P30101**	9.59	9.74	12.50	ER	56.8/5.98	Protein folding
11	**PDIA3**	**Protein disulfide-isomerase A3 precursor**	**P30101**	10.24	5.29	7.13	ER	56.8/5.98	Protein folding
12	**GELS**	**Gelsolin**	**P06396**	5.93	3.12	3.98	C, CK	85.7/5.90	Actin depolymerizer
13	**HSPB1**	**Heat shock protein beta-1**	**P04792**	1.94	-1.25	1.26	C, N	22.8/5.98	Stress response
14	**GANAB**	**Neutral alpha-glucosidase AB**	**Q14697**	1.15	-1.56	-1.09	ER, G	106.9/5.74	CH Metabolism
15	**ANXA1**	**Annexin A1**	**P04083**	1.56	1.72	1.90	C, N, CM	38.7/6.57	Signal transduction
16	**SEPT2**	**Septin-2**	**Q15019**	1.08	-1.51	-1.35	N	41.5/6.15	Cell cycle/division
17	**ENOA**	**Alpha-enolase**	**P06733**	1.04	1.91	1.89	C, CM	47.2/7.01	Glycolysis
18	**EF1G**	**Elongation factor 1-gamma**	**P26641**	-1.28	-1.85	-1.92	C	50.2/6.25	Protein synthesis
19	**TCPG**	**T-complex protein 1 subunit gamma**	**P49368**	-1.39	-1.54	-1.96	C	60.5/6.10	Protein folding
20	**DPYL2**	**Dihydropyrimidinase-related protein 2**	**Q16555**	-1.12	-1.45	-1.79	C	62.3/5.95	Metabolism
21	**SODM**	**Superoxide dismutase mitochondrial**	**P04179**	-2.5	-1.3	-4.35	MIT	24.7/8.35	Redox
22	**PGAM1**	**Phosphoglycerate mutase 1**	**P18669**	-1.33	-1.23	-1.54	C	28.8/6.67	Glycolysis
23	**TPIS**	**Triosephosphate isomerase**	**P60174**	-1.69	-1.49*	-1.72	C	26.7/6.45	Glycolysis
24	**ANXA2**	**Annexin A2**	**P07355**	3.44	6.80	5.12	EXC, CM	38.6/7.57	Trafficking
25	**AK1C2**	**Aldo-keto reductase family 1 member C2**	**P52895**	-2	-2.13	-3.22	C	36.7/7.13	Metabolism
26	**ENOA**	**Alpha-enolase**	**P06733**	-1.96	-1.37	-1.92	C, CM	47.2/7.01	Glycolysis
27	**UGDH**	**UDP-glucose 6-dehydrogenase**	**O60701**	-1.16	-2.08	-1.85	C	55.0/6.73	Metabolism
28	**ANXA2**	**Annexin A2**	**P07355**	2.69	3.06	2.82	EXC, CM	38.6/7.57	Trafficking
29	**PGK1**	**Phosphoglycerate kinase 1**	**P00558**	-1.14	-2.33	-2.32	C	44.6/8.30	Glycolysis
30	**ATPA**	**ATP synthase subunit alpha, mitochondrial**	**P25705**	-1.43	-2.17	-2.22	MIT	59.8/9.16	Respiration
31	**KPYM**	**Pyruvate kinase isozymes M1/M2**	**P14618**	-1.59	-2.44	-2.5	C	57.9/7.96	Glycolysis
32	**TAGL2**	**Transgelin-2**	**P37802**	1.31	-1.09	-1.43*	CK, CM	22.4/8.41	Structural
33	**PRDX1**	**Peroxiredoxin-1**	**Q06830**	1.67	-1.12	-1.09	C	22.1/8.27	Redox
34	**G3P**	**Glyceraldehyde-3-phosphate dehydrogenase**	**P04406**	-1.27	-2.04	-2.63	C, CM	36.1/8.57	Glycolysis
35	**ALDOA**	**Fructose-bisphosphate aldolase A**	**P04075**	-1.22	-1.79	-1.89	C	39.4/8.30	Glycolysis

^**§ **^Protein accession number according to SwissProt and TrEMBL databases.

^**‡ **^Average volume ratio vs IL-1β, quantified by PDQuest 7.3.1. software. * Protein altered less than 1.5-fold but with a significance level above 95% by the Student's t-test (*p*< 0.05).

** Predicted subcellular localization according to PSORTII program.

^**§§ **^Theoretical molecular weight (*M*_r_) and isoelectric point (p*I*) according to protein sequence and Swiss-2DPAGE database.

MIT, mitochondria; ER, endoplasmic reticulum; C, cytoplasm; CM, cell membrane; CK, cytoskeleton; G, Golgi apparatus; N, nucleus; EXC, extracellular matrix.

**Figure 2:**
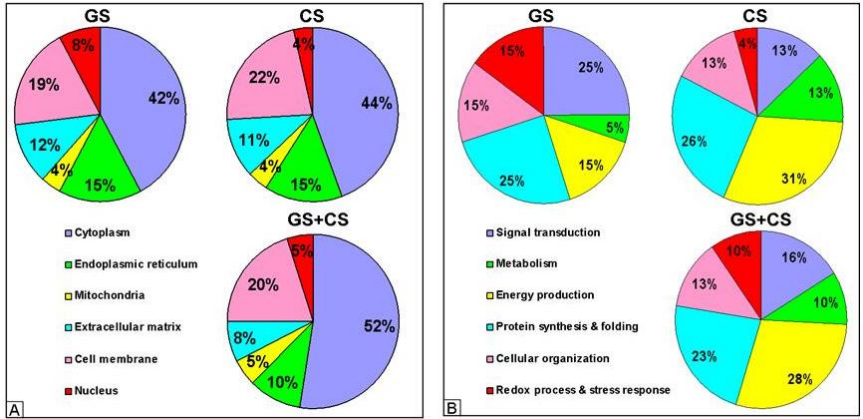
**Subcellular localization (A) and functional distribution (B) of the GS- and/or CS-modulated proteins identified by proteomics**. Database searches were used to classify these 35 proteins according to their subcellular localization and cellular function. Based on these characteristics, the proteins were assigned into six groups.

Database searches allowed us to classify these 35 proteins according to their subcellular localization and cellular function. Most of them (52%) were predicted to be cytoplasmic, while the remaining 48% were either associated with the cell membrane (20%), extracellular matrix (8%), or located in subcellular organelles, including the endoplasmic reticulum (10%), mitochondria (5%) or nucleus (5%) (Figure 2A). The predicted biological functions for these proteins fell into six major groups: 1) energy production; 2) signal transduction; 3) protein synthesis and folding; 4) redox process and stress response; 5) cellular organization; and 6) metabolism (Figure 2B).

### Proteins modulated by GS treatment

**Figure 3:**
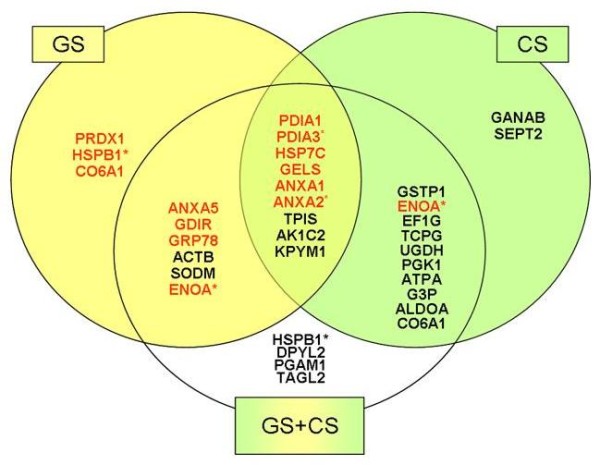
**Proteins modulated similarly and differently by GS-, CS- and GS+CS-treatment in IL-1β-treated human articular chondrocytes**. Proteins in the yellow circle are modulated by GS, proteins in the green circle are modulated by CS, and proteins in the white circle are modulated by the combination treatment. Upregulated proteins are indicated in red and downregulated proteins are in black (*two different isoforms; ^#^the same isoform).

We identified 18 different proteins that were modulated by GS (Figure 3). Fourteen of these proteins were increased compared to the control, while six were decreased. Three of these proteins were found to be positively modulated only by GS: peroxiredoxin-1 (PRDX1: redox process), HSPB1 (stress response) and collagen alpha-1(VI) chain precursor (CO6A1: cell adhesion). Most of the proteins increased by GS are involved in signal transduction pathways and in protein synthesis and folding processes (see Table 1). Interestingly, all the proteins modulated by GS treatment that are related to energy production were decreased; these include ENOA, triosephosphate isomerase (TPIS) and the pyruvate kinase isozymes M1/M2 (KPYM). Other pharmacological effects of GS involve the modulation of cellular organization processes (increase of gelsolin and decrease of actin) and redox and stress responses (decrease of mitochondrial superoxide dismutase).

### Proteins modulated by CS treatment

CS modulated 21 different proteins (Figure 3). Only nine proteins were increased, while 14 were decreased compared to the control condition. Interestingly CS, unlike GS, seems to affect mainly energy production and metabolic pathways. Proteins related to glycolysis represent the largest functional group decreased in chondrocytes treated with CS; these included glyceraldehyde 3-phosphate dehydrogenase (G3P), fructose biphosphate aldolase A (ALDOA), phosphoglycerate mutase 1 (PGAM1), TPIS, phosphoglycerate kinase 1 (PGK1), ATP synthase subunit alpha, mitochondrial (ATPA) and KPYM. Three metabolic proteins, AK1C2, GANAB and UDP-glucose 6-dehydrogenase (UGDH), were also decreased. Similar to GS treatment, many proteins modulated by CS are involved in protein synthesis and folding processes. Two proteins were modified only by CS, neutral alpha-glucosidase AB (GANAB), which is involved in glycan metabolism, and septin-2 (SEPT2), a cell cycle regulator (Figure 3).

### Proteins identified as modulated by GS and CS treatment

When administered in combination, GS and CS modified, in many cases, chondrocyte proteins synergistically. Overall, this combination modulated 31 spots corresponding to 29 different proteins, 12 of them were increased and 19 were decreased (Figure 3). These proteins are found in all the functional categories, but most are involved in energy production, protein synthesis and folding. Four of these proteins are modulated only by the combined treatment: a specific isoform of HSPB1, dihydropyrimidinase-related protein 2 (DPYL2), phosphoglycerate mutase 1 (PGAM1), and transgelin-2 (TAGL2).

### Verification of the modulation of GRP78 and SOD2

The results obtained by our pharmacoproteomic analysis need to be validated for differences in protein expression profiles before the biological roles of the modulated proteins are extensively studied. We selected two proteins, possibly involved in the OA process, on which to perform additional studies in order to verify their altered expression in GS and CS-treated chondrocytes: GRP78 and SOD2.

**Figure 4:**
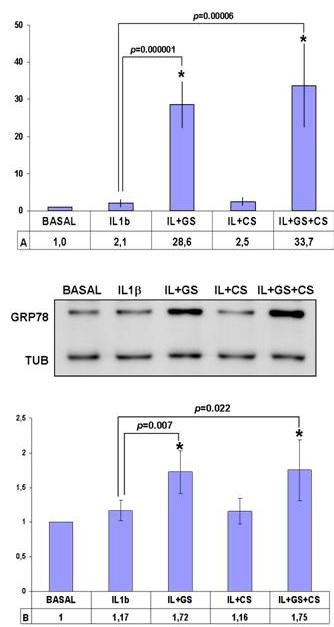
**The 78 kDa glucose-regulated protein precursor (GRP78) is increased by GS alone and in combination with CS. A**. Overexpression values of GRP78 determined by real-time polymerase chain reaction (PCR) analysis of cultured human articular chondrocytes treated with interleukin-1β (IL-1β) plus GS and/or CS (n = 6, *P *< 0.05*). **B**. Western blot analysis of GRP78 protein levels in treated chondrocytes. A representative blot is shown, along with the numeric data obtained by densitometry analysis of the blots (n = 4, *P *< 0.05*).

GRP78 was previously reported by our group to be related to OA pathogenesis [14]. We performed orthogonal studies to verify the eight-fold increase of this protein compared to the IL-1β-treated control group observed in the proteomic analysis. Real-time PCR assays demonstrated the GS-dependent upregulation of GRP78 gene expression, showing remarkable increases of almost 30-fold in GS-treated chondrocytes (*P *< 0.05, n = 6, age range: 55 to 63 years), and even slightly higher with combined GS and CS treatment (Figure 4A). These results were confirmed at the protein level by Western blot analysis in four independent experiments. Densitometric analysis of the band intensities revealed an increase of GRP78 protein in GS- and GS + CS-treated samples that averaged 1.72-fold and 1.75-fold greater than control (*P *< 0.05) (Figure 4B).

**Figure 5:**
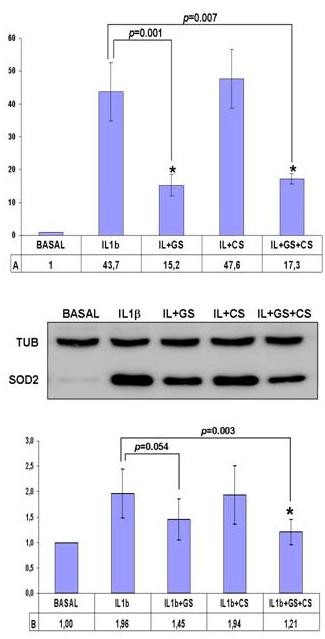
**Mitochondrial superoxide dismutase (SOD2) is decreased by GS alone and in combination with CS. A**. Underexpression values of SOD2 determined by real-time polymerase chain reaction (PCR) analysis on cultured human articular chondrocytes treated with interleukin-1β (IL-1β) plus GS and/or CS (n = 4, *P *< 0.05*). **B**. Western blot analysis of SOD2 protein levels in treated chondrocytes. A representative blot is shown, along with the numeric data obtained by densitometry analysis of the blots (n = 7, *P *< 0.05*).

Mitochondrial SOD2, a protein previously reported to be related to the OA disease process [26], was decreased by GS and GS+CS treatment in our proteomic screening. To validate our data, real-time PCR analyses were carried out on RNA samples isolated from four independent experiments (Figure 5A). The results showed a significant (*P *< 0.001) up-regulation of SOD2 gene expression in IL-1β-stimulated cells, with an increase of 44-fold, and a subsequent 70% decrease in GS- and GS+ CS-treated cells. We also carried out Western blot analyses to examine SOD2 modulation at the protein level. A decrease in SOD2 protein levels was evident in all donors (n = 7, age range: 51 to 72 years old). Figure 5B shows data from the densitometric analysis of the blots, revealing a two-fold increase in IL-1β-stimulated cells with subsequent 75% decrease of SOD2 in GS + CS-treated cells (*P *< 0.05).

## Discussion

In the present work, we examined the utility of a pharmacoproteomic approach for analyzing the putative intracellular targets of glucosamine (GS) and chondroitin sulphate (CS) in cartilage cells. Using proteomic techniques, we studied the influence of these compounds, both alone and in combination, on the molecular biology of chondrocytes challenged with the proinflammatory cytokine IL-1β.

The conditions used in this study represent supraphysiological levels of both drugs and cytokine. These concentrations, however, are included in the range of *in vitro *concentrations used by other laboratories, thus facilitating the comparison with other studies [27,28]. In our work, we chose them according to the bibliography, where a very wide range of both glucosamine and chondroitin sulfate have been used on different cell types and tissues [29,30]. We tested different concentrations of both drugs in the standardization step of the proteomic analysis (CS from 10 to 200 μg/ml and GS from 1 to 10 mM), and selected the highest concentrations in order to better unravel the molecular mechanisms that are modulated by these compounds. Moreover, in the case of glucosamine it is important to emphasize that its pharmacokinetic is modulated by the levels of glucose in the culture medium, as it utilizes glucose transporters to be taken up by the cells [31,32]. Since our cells are grown under high levels of glucose (DMEM, containing 25 mmol/l glucose), it is necessary to use high concentrations of glucosamine in order to appreciate its effect in the presence of high glucose. The molecular mechanisms driven with these high amounts of both drugs might not be comparable to their classical oral administration, but they can mimic a direct delivery into the joint. In this sense, it has been recently proposed that intra-articular administration of CS may provide an immediate contact with the synoviocytes and chondrocytes, as is the case in cellular culture models [33]. Furthermore, a recent study performed on cartilage explants shows how cyclic preloading significantly increased tissue PG content and matrix modulus when they are directly supplemented with high concentrations of the combination of GS and CS (500 μg/ml and 250 μg/ml, respectively), resulting in a reduction of matrix damage and cell death following an acute overload [34].

All the mentioned limitations are inherent to *in vitro *studies, and also highlight the screening utility of proteomic approaches. Given the high complexity of these kinds of studies (and specifically the present one, in which five different conditions are evaluated), it is essential to be reminded how these approaches aim to screen for differences between the conditions that are being compared, opening the door for subsequent more exhaustive verification studies of some of these changes (which would allow both the inclusion of more samples to be analyzed and the performance of time-course or dose-response experiments). As a proof of the act, in this work (and based on their previously described relationship with OA pathogenesis) we selected one protein that was increased (GRP78) by the drug treatment and one that was decreased (SOD2), and performed orthogonal studies on them to verify their alteration.

Despite their limitations, several *in vitro *studies have previously shown how CS and GS could moderate some aspects of the deleterious response of chondrocytes to stimulation with IL-1β. In chondrocyte cultures, GS and CS diminish the IL-1β-mediated increase of metalloproteases, [35,36] the expression of phospholipase A2 [37,38] and cyclooxygenase-2, [39] and the concentrations of prostaglandin E_2 _[40]. They also reduce the concentration of pro-inflammatory cytokines, such as tumor necrosis factor-α (TNF-α) and IL-1β, in joints, [41] and systemic and joint concentrations of nitric oxide [42] and reactive oxygen species (ROS) [43]. All these studies showed similar results for both molecules, mainly related to their anti-inflammatory effect, while the results obtained by our pharmacoproteomic approach highlight the different molecular mechanisms affected by GS or CS. It is essential to point out that our study has been performed with chondrocytic intracellular extracts. In this context, it is difficult to identify proteins that are known to be secreted by the chondrocytes, such as metalloproteinases, cytokines or aggrecanases, which have been the focus of a recent mRNA-based analysis [44], or hyaluronan synthases, which have been newly found to be increased by CS in synoviocytes [45]. All these were also described to be modulated by GS in a previous transcriptomic study [10]. However, detection of this type of proteins in intracellular fractions by shotgun proteomics is not easily achievable because they are mainly delivered to the extracellular space after their synthesis, being those small amounts that are retained inside the cells masked by other typical cytoplasmic proteins which are more abundant [13]. Given the high dynamic range of proteins in biological systems, this problem is inherent to global screening proteomic experiments, and is only solvable employing hypothesis-driven proteomics strategies (targeted proteomics).

**Figure 6:**
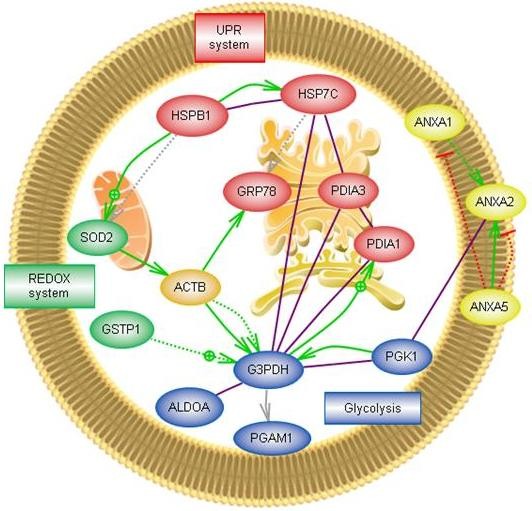
**Pathways and networks related to chondrocyte proteins identified by proteomics as altered by GS and/or CS**. Pathway Studio software was used to map the identified proteins into characterized human pathways and networks that associate proteins based on known protein-protein interactions, mRNA expression studies and other previously described biochemical interactions. Abbreviations are shown as in Table 1. Most of the proteins modulated by GS belong to the unfolded protein response (UPR) system, while CS seems to affect mainly energy production (glycolysis) and metabolic pathways.

As mentioned before, this study is focused on the investigation of the intracellular mechanisms modulated by CS and GS, which are the background for ulterior putative changes of ECM turnover. In our work, 25% of the proteins modulated by GS are involved in signal transduction pathways, 15% in redox and stress response, and 25% in protein synthesis and folding processes, whereas CS affects mainly energy production (31%) and metabolic pathways (13%) by decreasing the expression levels of 10 proteins (Figure 1B). Bioinformatic analysis using Pathway Studio 6.1 software (Ariadne Genomics, Rockville, MD, USA) enabled the characterization of the biological association networks related to these differentially expressed chondrocytic proteins. A simplified picture of their interactions is showed in Figure 6. Using this analysis, we identified the biochemical pathways that may be altered when chondrocytes are treated with GS and CS.

Most of the proteins modulated by GS belong to the complex homeostatic signalling pathway known as the unfolded protein response (UPR). The UPR system is involved in balancing the load of newly synthesized proteins with the capacity of the ER to facilitate their maturation. Dysfunction of the UPR plays an important role in certain diseases, particularly those involving tissues like cartilage that are dedicated to extracellular protein synthesis. The effect of GS on molecular chaperones and the role of protein disulfide isomerases (PDIs) in the maturation of proteins related with cartilage ECM structure have been described [46]. PDIA3 (GRP58) is a protein on the ER that interacts with the lectin chaperones, calreticulin and calnexin, to modulate the folding of newly synthesized glycoproteins [47], whereas PDIA1 (prolyl 4-hydroxylase subunit beta) constitutes a structural subunit of prolyl 4-hydroxylase, an enzyme that is essential for procollagen maturation [48]. The marked GS-mediated increase of these proteins in chondrocytes points to an elevation in ECM protein synthesis, which might be also hypothesized by the detected increase in Type IV Collagen (COL6A1, essential for chondrocyte anchoring to the pericellular matrix [49]) synthesis caused by GS.

Finally, GS remarkably increases another UPR-related protein, GRP78 (BiP), a fact that we confirmed both at transcript and protein levels. This protein is localized in the ER, and has been previously identified as an RA autoantigen [50], which was subsequently characterized by its anti-inflammatory properties through the stimulation of an anti-inflammatory gene program from human monocytes and the development of T-cells that secrete regulatory cytokines such as IL-10 and IL-4 [51]. In a previous work, we found an increase of this protein in OA chondrocytes, which might be a consequence of heightened cellular stress [14]. A number of previous reports have described the positive modulation of GS on ER proteins, including GRP78 expression [52], but this is the first time that such modulation was found to arise from GS treatment in chondrocytes; thus interestingly suggesting an specific mechanism of action for the putative anti-inflammatory effect of GS in OA.

On the other hand, most proteins modulated by CS are proteins related to metabolism and energy production. It is remarkable that all except one (an enolase isoform) were decreased. In this group, we identified seven out of the 10 enzymes that directly participate in the glycolysis pathway (aldolase, triose phosphate isomerase, glyceraldehyde phosphate dehydrogenase, phosphoglycerate kinase, phosphoglyceromutase, enolase and pyruvate kinase). This suggests that, while IL-1β treatment tends to elevate glycolytic energy production ([15] and our observations), it is then lowered by CS (which reduces five of these enzymes) and by the combination of both drugs (which reduces all seven glycolytic enzymes). The decrease of Neutral alpha-glucosidase AB (or glucosidase II, GANAB), only caused by CS alone (Figure 2), and two other metabolism-related proteins (AK1C2 and UGDH), points also to a reduction of cellular metabolism. GANAB is an ER-enzyme that has profound effects on the early events of glycoprotein metabolism, and has been recently proposed as biomarker for detecting mild human knee osteoarthritis [53].

Interestingly, only four proteins were found to be modulated by GS and CS combination but not by either of the drugs alone, whereas we observed a quantitative synergistic effect of the combination in more than a half (55%) of the altered proteins (Table 1). One of the proteins whose decrease by both drugs alone was significant and furthermore powered by their combination is the redox-related protein SOD2. This protein, the mitochondrial superoxide dismutase, has substantial relevance in stress oxidative pathways and in cytokine-related diseases, such as OA [54]. We found SOD2 to be upregulated by IL-1β ([15] and our observations), and downregulated by GS and CS treatment, both at the transcriptional and protein levels (Real Time-PCR and Western blotting). Supporting our findings, other authors have recently reported the role of GS in counteracting the IL-1β-mediated increase of inducible nitric oxide synthase (iNOS) and the decrease of heme oxygenase, and indicated that the influence of GS and CS on oxidative stress is a possible mechanism of action for its protective effect on chondrocytes [55].

## Conclusions

Taking into account the limitations of an *in vitro *study, our findings provide evidence for the usefulness of proteomics techniques for pharmacological analyses. The potential application of this approach is to identify efficacy markers for monitoring different OA treatments. In this study, a number of target proteins of GS and CS have been described, pointing out the wide-ranging effects of these drugs on fundamental aspects of chondrocyte metabolism, but also their alternative mechanisms of action in a system model of OA.

## Abbreviations

2-DE: two-dimensional electrophoresis; cDNA: complementary DNA; CS: chondroitin sulfate; C_t_: threshold cycle; DMEM, Dulbecco's modified Eagle's medium; ECM: extracellular matrix; ER: endoplasmic reticulum; FCS: fetal calf serum; GRP78: glucose regulated protein 78; GS: Glucosamine sulfate; IEF: isoelectric focusing; IL-1β: inteleukin-1β; IPG: immobilized pH gradient; MALDI-TOF: Matrix-assisted laser desorption/ionization Time of Flight; MS: mass spectrometer; OA: osteoarthritis; PCR: polymerase chain reaction; SDS: sodium dodecyl sulphate; SOD2: superoxide dismutase 2; SYSADOA: Symptomatic Slow-Acting Drugs for Osteoarthritis; UPR: unfolded protein response.

## Competing interests

EM is the Head of the Medical Area from Bioibérica, SA. JV is Medical Director of Bioibérica, SA. FJB received a grant from Bioibérica, SA to carry out this project. The authors declare no other competing interests.

## Authors' contributions

VC carried out the experimental work, analysed the data and drafted the manuscript. CRR participated in study design, interpretation of data and manuscript preparation. BR helped in collecting and processing protein samples, participated in Western blot experiments and helped in statistical data analysis. PFP and JM carried out the mass spectrometry analysis and database search. LM and JV provided CS and GS and helped design the study. FJB conceived and coordinated the project and revised the manuscript. All authors read and approved the final manuscript.
